# Bioconversion of *p*-Tyrosol into Hydroxytyrosol under Bench-Scale Fermentation

**DOI:** 10.1155/2018/7390751

**Published:** 2018-07-09

**Authors:** Zouhaier Bouallagui, Sami Sayadi

**Affiliations:** Laboratory of Environmental Bioprocesses, Centre of Biotechnology of Sfax, University of Sfax, P.O. Box “1177”, Sfax 3018, Tunisia

## Abstract

Tyrosol hydroxylating* Pseudomonas *strain was previously isolated from olive mill wastewaters-irrigated soil. In the present work, experimental design was used to study the bioconversion of tyrosol in laboratory fermenters aiming at the recovery of the highest yields of hydroxytyrosol. The effects of biocatalyst loading and tyrosol concentration were studied. The bioconversion yield reached 86.9% (37.3 mM hydroxytyrosol) starting from a tyrosol concentration of 43 mM. Under these conditions, the specific productivity relative to the biocatalyst was 4.78 *μ*M/min/g. The established model to predict bioconversion yield was validated in two bench-scale fermenters. At the downstream stage, the reaction product was recovered as a hydroxytyrosol rich solution after microfiltration and concentration under vacuum. Subsequent to this operation, hydroxytyrosol composition yielded 73.8% of the total dry matter.

## 1. Introduction

The bioactive properties of hydroxytyrosol have triggered the establishment of a new insight into its use as a naturally available antioxidant, mostly found in olive tree [1, 2]. This later is thought to be a substitute to synthetic antioxidants in foods [3]. Indeed, this compound was demonstrated to prevent lipid peroxidation in many* in vitro* and* in vivo *models [4–6]. Synthetic pathways in the olive tree do not lead to the direct synthesis of hydroxytyrosol. Yet, this later mainly results from oleuropein hydrolysis taking place during olive fruit maturation [7]. Besides, this hydrolysis will be enhanced during the olive oil extraction process resulting in the increase in hydroxytyrosol concentration in olive mill wastewater, due to its low oil/water partition coefficient [8, 9]. This fact, associated with studies showing the beneficial activities of hydroxytyrosol, has promoted the development of efficient hydroxytyrosol recovery processes.

Despite the increasing demand for the use of hydroxytyrosol as a nutraceutical, there are still limited recovery processes that could be succeeded. Furthermore, although successful, these processes cannot satisfy the market requirements. Relevant recovery processes have used olive mill wastewater as a natural resource of hydroxytyrosol [10]. These processes were based on solvent extraction procedures. Two major drawbacks could be assigned for such procedures, which are the risk of solvent contamination and the need for additional chromatographic purification steps. The continuous countercurrent extraction system was suggested as the choice to partially overcome these problems [11]. However, the production yields were considered as unsatisfying.

During the last decade, few relevant processes based on bioconversion reactions were developed in order to produce hydroxytyrosol. Enzymatic processes using tyrosinase or *β*-glucosidase as biocatalysts and tyrosol or oleuropein as respective substrates have been reported [12–15]. In both cases, the purification of the enzyme rises as a major drawback. In addition, the heterogeneity of the reaction products or the use of a cofactor as ascorbic acid will further affect the product cost. Yet, a few numbers of researchers have reported the use of entire bacterial cells as effective catalysts for the recovery of biolabeled hydroxytyrosol. Bacterial strains belonging to* Pseudomonas *[16],* Serratia *[17], transformed* E. coli* [18], or* Halomonas* [19] strains were earlier identified as being capable of transforming tyrosol to hydroxytyrosol. The present work was designed to understand the laboratory fermentation process for the bioconversion of tyrosol into hydroxytyrosol through the elaboration of an experimental design model involving the substrate and biomass concentrations as key variables that could influence the reaction using a bacterial strain isolated by our team.

## 2. Materials and Methods

### 2.1. Inoculum Preparation

*Pseudomonas aeruginosa* strain, previously isolated and identified in our laboratory [16], was cultivated in Lauria broth in one litre Erlenmeyer flasks inoculated from solid media (1.5% agar). For the induction of tyrosol metabolizing pathway, filter sterilized tyrosol solution was added to the culture at a final concentration of 0.6 g/L. The culture was then allowed to grow overnight at 30°C with orbital shaking at 180 rpm.

### 2.2. Biomass Production

The biomass used for the conversion of tyrosol into hydroxytyrosol was grown in mineral medium (containing in g/L): Na_2_HPO_4_, 2.44; KH_2_PO_4_, 1.52; (NH_4_)_2_SO_4_, 1.5; MgSO_4_, 7H_2_O, 0.2; CaCl_2_, H_2_O, 0.05 and 10 ml of a trace-element solution containing ( in g/L): EDTA, 0.5; FeSO4, 0.2; ZnSO_4_, 7H_2_O, 0.01; MnCl_2_, 4H_2_O, 0.003; Na_2_MoO_4_, 2H_2_O, 0.003; H_3_BO_3_, 0.03; CuCl_2_, 6H_2_O, 0.002; NiCl_2_, 6H_2_O, 0.002; and CoCl_2_, 6H_2_O, 0.02. The pH of the medium was adjusted to 7.2. Tyrosol was used at 1 g/L as a unique carbon source. Two fermentation types (3.6 L and 7.5 L fermenters, INFORS AG CH-4103 Bottmingen/Switzerland) were inoculated with the induced inoculum. The biomass was grown at 30°C and the agitation and the aeration in the fermenters were adjusted in order to keep the oxygen saturation rate in the range of 30%. Bacterial growth was followed spectrophotometrically until the late logarithmic phase. At this stage, the culture was collected by centrifugation (5500×g at 4°C for 10 min) and washed twice with M9 phosphate buffered saline (4.2 mM Na_2_HPO_4_, 2.2 mM KH_2_PO_4_, 0.9 mM NaCl, and 1.9 mM NH_4_Cl). The biomass was again resuspended in M9 phosphate buffered saline as bioconversion medium. Final biomass and tyrosol concentrations were chosen following the experimental design (Table 1). All runs were performed in 2 L working volume (3.6 L fermenter).

### 2.3. Experimental Design and Optimization

Response surface methodology using a central composite design was adopted to determine the effect of biomass and substrate on the production of hydroxytyrosol. Three coded values were fixed for each of the continuous variables. These coded values were calculated according to the equation

(1) $$\begin{array}{c} x_{i}=\frac{X_{i}-X_{0}}{\Delta X} \end{array}$$

*x*_*i*_ is the coded value of the independent variable, *X*_*i*_ the natural value of the independent variable, *X*_0_ the natural value of the independent variable at the centre point, and Δ*X* the step change value (Δ*X* is 1.25 for the biomass concentration and 1 for the substrate concentration). A set of 18 experiments was generated by the experimental design (Table 1).

The response (hydroxytyrosol production (mM)) was correlated with the independent variables (biomass and tyrosol concentrations) through a second-order polynomial equation:

(2) $$\begin{array}{c} Y=b_{0}+b_{1}x_{1}+b_{2}x_{2}+b_{11}x_{1}^{2}+b_{22}x_{2}^{2}+b_{12}x_{1}x_{2} \end{array}$$

where *Y* is the hydroxytyrosol production (mM); *x*_1_ and *x*_2_ are the coded values of the biomass and tyrosol concentration, respectively. b_0_ is the intercept; b_1_ and b_2_ are linear coefficients for biomass and tyrosol, respectively; b_11_ and b_22_ are squared coefficients and b_12_ is the interaction coefficient. Data were analyzed using JMP 9.0.2 software.

### 2.4. Analytical Method

Throughout the bioconversion time, samples (1 mL) were withdrawn periodically (every 2 hours or less depending on the progress of the reaction) and centrifuged at 8000×g for 10 min. The supernatant was analyzed using high performance liquid chromatography (HPLC) to determine the concentration of the different metabolites. The instrument consisted of a Shimadzu C-R6A liquid chromatograph coupled to a Shimadzu SPD-6A UV detector. The separation was carried out on a C18 reverse phase column (250 mm×4.6 mm; 5 *μ*m particle size; Shim-pack VP-ODS). The mobile phase consisted of (A) 0.1% formic acid in water and (B) 70% acetonitrile in water. The following gradient was used: 0 min 10% B, 25 min 25% B, 35 min 80% B, 37 min 100% B, 40 min 100% B, and 50 min 10% B. The flow rate was set at 0.7 mL/min. The injection volume was 20 *μ*L. The eluted compounds were detected at *λ* 280 nm and identified by comparison with authentic standards.

## 3. Results and Discussion

### 3.1. Predictive Model of Hydroxytyrosol Production

**Figure 1:**
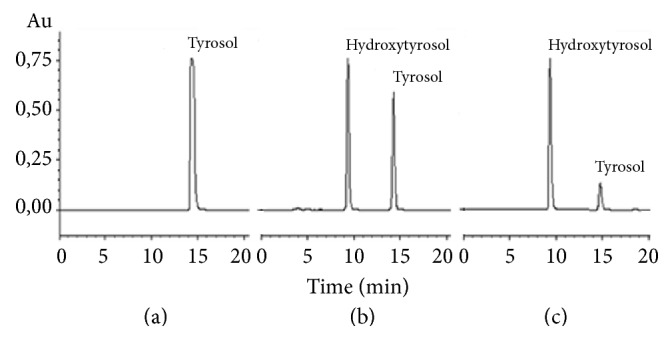
Illustrative time course chromatograms for the bioconversion of tyrosol into hydroxytyrosol. (a) HPLC spectrum of bioconversion medium at starting time; (b) composition of reaction medium at reaction mid-time; (c) composition of bioconversion medium at a late stage of the reaction.

We previously studied the bioconversion of tyrosol into hydroxytyrosol in Erlenmeyer flasks [16]. In the present work, we are focusing on the scale-up of the reaction into laboratory fermenters. In order to examine the hydroxytyrosol production (mM), two independent variables, biomass and tyrosol concentrations, were investigated using a response surface methodology. The biomass and tyrosol concentrations were varied in the ranges of 2.5 to 5 g (wet weight)/L and 4 to 6 g/L (28.9 to 43.4 mM), respectively. These two variables were investigated through the use of a central composite design. The experimental design generated 18 experiments among which only 16 runs were considered and the two others were discarded due to an excessive lengthening of the reaction time (Table 1). For all of the sixteen experiments, reaction time ranged between 18 and 27 hours. However, for experiments 17 and 18, reaction times lasted for more than 50 hours. Table 1 shows that the highest hydroxytyrosol production was obtained at 5 g/L biomass and 6 g/L tyrosol (experiment N° 2). Under these working concentrations, the molar bioconversion yield was brought to 86.11% with 37.39 mM hydroxytyrosol production. A representative time course evolution of the bioconversion reaction is given in Figure 1.

**Figure 2:**
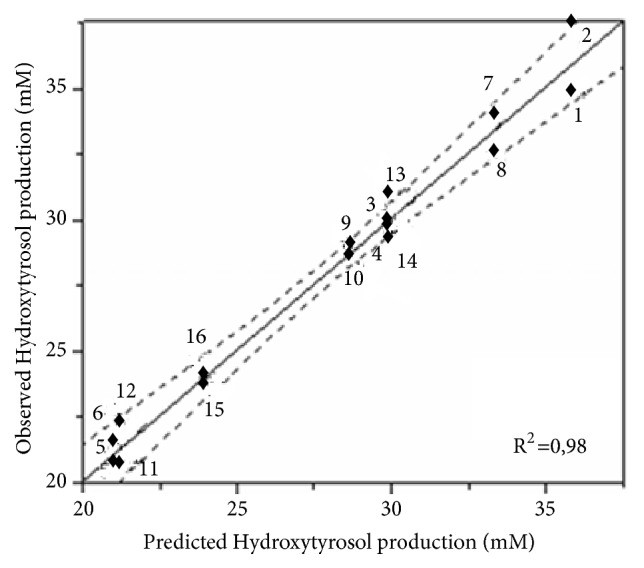
Correlation between experimental and predicted response values for hydroxytyrosol production. Numbers correspond to the experiment number given in Table 1. Dashed line: 0.05 significance curve. Continuous line: line of fit.

The analysis of variance (ANOVA) and the lack of fit of the predictive model are shown (Table 2). Analysis was based on* p* values as a criterion in order to check the significance of the model [20, 21]. The model F-value of 117.235 associated with a* p* value <0.0001 implies that the model is highly significant. In fact, this probability denotes that the noise error is less than 0.01%. Another marker of the goodness of our model is the regression coefficient* R*^*2*^. A coefficient *R*^2^ = 0.983 indicates that only 1.7% of the experimental data cannot be explained by the predicted model (Figure 2). The lack of fit (0.326) with a probability of 0.730 implies that it is insignificant relatively to the pure error.

The calculated coefficient values of the predicted model and their significance are shown in Table 3. Through the analysis of the* p* values it is shown that the biomass linear coefficient (*b*_*1*_) is not significant either at 1% or at 5%. Nevertheless, for the same variable, the quadratic (*b*_*11*_) and the cross-product (*b*_*12*_) coefficients are still significant at 5%. Furthermore, the substrate linear coefficient (*b*_*2*_) is highly significant at 1%. Finally, the quadratic coefficient* b*_*22*_ seems to be insignificant at 1% confidence interval. These data suggest that the biomass loading does not have a significant effect on hydroxytyrosol production. Nonetheless, biomass concentration does have an effect only on the reaction time (data not shown). This fact was understood after calculating the second order polynomial model, which could clearly explain the effect of the studied variables on hydroxytyrosol production expressed in the following equation:

(3) Y=−3.729−11.895X1+15.917X2+0.817X12−1.426X22+1.151X1X2

where *Y* represents the hydroxytyrosol production and X_1_ and X_2_ are biomass and tyrosol concentrations (g/L), respectively.

### 3.2. Experimental Model Validation

For the model validation, a couple of biomass and tyrosol concentrations (g/L) was chosen (4.25; 5.5). For these concentrations, the model predicted a hydroxytyrosol production of 31.81 mM. In order to validate the model, two duplicated experiments were run in two different fermenters (3.6 L and 7.5 L). Under these conditions, the experimental hydroxytyrosol production values were 32.16 and 31.08 mM in both fermenters, respectively. The corresponding reaction times for both fermenters were 24.33 and 25.25 hours. Therefore, these data clearly show that the reaction times for the validation experiments are still within the range of all other experiments (18 to 27 hours) and the experimental hydroxytyrosol production values are in agreement with those predicted by the model.

Despite the biological importance of hydroxytyrosol as a potent antioxidant molecule, which might be implicated in the prevention of oxidative stress related diseases, its recovery is still challenging. Processes including chemical synthesis and recovery from olive by-products (leaves or milling wastewater) are not without limitations [10, 22]. Recently, several biological processes have been reported. These bioprocesses include either enzymatic or microbial catalysts [16, 17, 19, 23]. Within the present work, we showed that the bacterial recovery of hydroxytyrosol could be interesting considering the highest achievable yield and the scale-up feasibility of the bioconversion process.

### 3.3. Hydroxytyrosol Recovery

The recovery and probably the purification of the bioconversion product are considered as ultimate stages in the reaction process. The majority of the available hydroxytyrosol recovery processes are based on chromatography purification either from olive by-products or biological (enzymatic or cellular) and chemical reactions. Within the present process, the reaction product was recovered after the first step of centrifugation at 8000×g for 10 minutes, followed by a microfiltration (0.22 *μ*m) in order to eliminate any traces of microorganisms. The filtrate was then concentrated under vacuum using a rotatory evaporator. A 60-fold concentrated product was obtained. This step will allow the precipitation of a major part of the salts present in the reaction medium. Subsequently, characterization by HPLC for qualitative and quantitative analysis of hydroxytyrosol and other potential metabolites was performed. Results showed that the concentrated solution contained 16.7% (w/v) hydroxytyrosol, which was equivalent to 73.8% of the total dry matter. To our knowledge, this composition could offer the highest percentage of biolabeled hydroxytyrosol yet available on the market. Thereafter, a single chromatographic step or the application of membranes would allow the recovery of high purity hydroxytyrosol. In particular, the use of membrane technology would result in an efficient selectivity towards target molecules. For this reason, membranes were the option when purifying hydroxytyrosol from olive mill wastewaters [24–26].

## 4. Conclusion

Considering the above shown data, it can be advanced that the fermentation process adopted for the bioconversion of* p*-tyrosol into hydroxytyrosol might be encouraging for transferring this reaction to the pilot scale and potentially using a safer engineered microorganism. In addition, response surface methodology will always be a prosperous approach to investigate variables interactions when many are considered.

## Figures and Tables

**Table 1:** Experimental design and results of the bioconversion of tyrosol into hydroxytyrosol. Numbers between parentheses represent the coded values of both studied variables.

**Experiment**	**Biomass (g/L)**	**Tyrosol ** **(g/L)**	**Hydroxytyrosol production (mM)**		**Residual hydroxytyrosol production ** **(mM)**
			**Observed**	**Predicted**	
1	5.00 (+1)	6.00 (+1)	34.91	35.92	-1.017
2	5.00 (+1)	6.00 (+1)	37.39	35.92	1.462
3	5.00 (+1)	5.00 (0)	29.88	29.93	-0.056
4	5.00 (+1)	5.00 (0)	29.48	29.93	-0.454
5	5.00 (+1)	4.00 (-1)	20.75	21.09	-0.343
6	5.00 (+1)	4.00 (-1)	21.50	21.09	0.408
7	3.75 (0)	6.00 (+1)	33.61	33.22	0.387
8	3.75 (0)	6.00 (+1)	32.39	33.22	-0.832
9	3.75 (0)	5.00 (0)	28.87	28.67	0.195
10	3.75 (0)	5.00 (0)	28.61	28.67	-0.064
11	3.75 (0)	4.00 (-1)	20.66	21.27	-0.614
12	3.75 (0)	4.00 (-1)	22.20	21.27	0.928
13	2.50 (-1)	5.00 (0)	30.9	29.96	0.934
14	2.50 (-1)	5.00 (0)	29.41	29.96	-0.555
15	2.50 (-1)	4.00 (-1)	23.73	24.00	-0.274
16	2.50 (-1)	4.00 (-1)	23.9	24.00	-0.104
17	2.50 (-1)	6.00 (+1)	-	-	-
18	2.50 (-1)	6.00 (+1)	-	-	-

**Table 2:** Analysis of variance (ANOVA) for the predicted quadratic model.

Source	Sum of squares	DF	Mean square	F-value	Probability *p*
Model	414.02	5	82.804	117.23	<.0001
Lack of fit	0.53	2	0.266	0.326	0.73
Pure error	6.530	8	0.816		
Corrected total	421.08	15			

**Table 3:** Estimated values of the model coefficients.

Model coefficients	Estimate	Standard Error	*P*-value
*b*_*0*_	28.674	0.467	<.0001
*b*_*1*_	-0.012	0.330	0.9697
*b*_*2*_	5.974	0.330	<.0001
*b*_*12*_	1.439	0.449	0.0094
*b*_*11*_	1.277	0.476	0.0230
*b*_*22*_	-1.426	0.476	0.0135

## Data Availability

The data used to support the findings of this study are available from the corresponding author upon request.
